# Node property of weighted networks considering connectability to nodes within two degrees of separation

**DOI:** 10.1038/s41598-018-26781-y

**Published:** 2018-05-31

**Authors:** Shun-ichi Amano, Ken-ichiro Ogawa, Yoshihiro Miyake

**Affiliations:** Department of Computer Science, Tokyo Institute of Technology, Yokohama, Kanagawa Japan

## Abstract

Weighted networks have been extensively studied because they can represent various phenomena in which the diversity of edges is essential. To investigate the properties of weighted networks, various centrality measures have been proposed, such as strength, weighted clustering coefficients, and weighted betweenness centrality. In such measures, only direct connections or entire network connectivity from arbitrary nodes have been used to calculate the connectivity of each node. However, in weighted networks composed of autonomous elements such as humans, middle ranges from each node are also considered to be meaningful for characterizing each node’s connectability. In this study, we define a new node property in weighted networks to consider connectability to nodes within a range of two degrees of separation, then apply this new centrality to face-to-face human communication networks in corporate organizations. Our results show that the proposed centrality distinguishes inherent communities corresponding to the job types in each organization with a high degree of accuracy. This indicates the possibility that connectability to nodes within two degrees of separation reveals potential trends of weighted networks that are not apparent from conventional measures.

## Introduction

Network analysis is a useful method for analysing the structure of many-body systems from a topological viewpoint^1^. In this form of analysis, a many-body system is mathematically represented by a simple network composed of elements (nodes) and connections (edges) between elements. This method has revealed well-known structural features of complex networks such as the small-world property and the scale-free property^2,3^. In addition, not only the topology of weighted networks, but also the diversity of their connections has been analysed. In weighted networks, each edge has additional information called ‘weight’. Weight is important for investigating many interesting phenomena emerging from such networks. For example, in real weighted networks such as traffic networks, brain networks, and social networks, weight represents the number of commuters between towns, the magnitudes of correlational interactions between brain regions, and the intimacy between humans, respectively^4–6^.

Various centrality measures (henceforth “centralities”) for weighted networks have been proposed to investigate the properties of weighted networks, for example strength, weighted clustering coefficients, and weighted betweenness centrality^7–9^. When such centralities are included in the analysis, it becomes possible to reveal the relationship between weights and topology. One typical form of analysis investigates the relationship between degree^10^ and strength, each of which represents the number of the edges connected to each node and the sum of the weights assigned to them. This relationship reveals interesting trends in weighted networks. In particular, power–law correlations between degree and strength have been observed in many weighted networks in real social systems^7,8,11,12^. Furthermore, various mathematical models of network dynamics have been proposed to explain the mechanisms whereby correlations emerge^13–16^.

**Figure 1:**
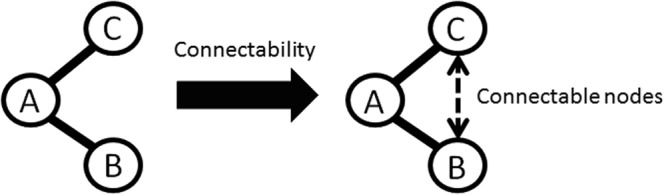
The triangular relationship between persons A, B, and C. In the relationship between persons A, B, and C, if there are strong ties between A and B, and between A and C, a tie between B and C will be established. In this situation, A and C are connected with two degrees of separation. According to this assertion, connectability between two nodes with two degrees of separation should be considered in this structural relationship.

In these centralities for weighted networks, only direct connections or entire network connectivity from arbitrary nodes have been used to calculate the connectivity of each node. However, this limitation may be too strict for analyses of weighted networks composed of autonomous elements, as humans are. From a topological viewpoint, each element in such networks can easily connect to other elements in the area beyond the nearest neighbour. On the other hand, no single node predetermines the structure of network connections over the entire network. Hence, middle ranges from each node, rather than direct connections or entire network connectivity, are considered to be meaningful for characterizing each node’s connectability. For example, consider a relationship between three people, A, B, and C. If person A is a common friend of B and C, then person B has a high probability of encountering and communicating with person C. This possibility has already been dubbed the ‘forbidden triad’ by Granovetter in social science. That is, in the relationship between persons A, B, and C, if there are strong ties between A and B, and between A and C, then a tie between persons B and C will be established^17^. Such a relationship is typical of phenomena that are never revealed by studies focusing only on direct connections between elements. According to Granovetter’s theory, connectability between nodes within two degrees of separation, hereafter called ‘easily connectable nodes’, has an important meaning in human communication networks (Fig. 1). Therefore, we expect to reveal potential trends or relationships in weighted networks by considering the connectability between nodes within two degrees of separation. However, conventional centralities for weighted networks do not explicitly quantify this form of connectability for each node.

In this study, we propose a new property of weighted networks to consider the connectability of each node to others within two degrees of separation, based on the node’s allocable weight (resource). Using this new centrality, we analyse human face-to-face communications in social organizations as an example of weighted networks composed of autonomous elements. Finally, we verify that this centrality can successfully identify characteristics of departments in these organizations.

## Results

### Node Connectivity to Others with One Degree of Separation

Let us briefly summarize the mathematical notation for weighted networks with *N* nodes. ***W***(=[*w*_*ij*_]) is called a ‘weighted adjacency matrix’ with *N* × *N* elements, in which *w*_*ij*_ = 0 when there is no edge between nodes *i* and *j* but *w*_*ij*_ = *w* when an edge exists, where *w* is a real number. In this matrix, if *w* = 1, ***W*** is reduced to an adjacency matrix ***A***(=[*a*_*ij*_]) where *a*_*ij*_ = 0 or 1. If we focus only on the presence or absence of edges in a weighted network, we can use ***A*** instead of ***W***.

To quantify node connectability within the range of two degrees of separation, we consider each node’s allocable weights. Specifically, the allocable weights can be defined as reallocation of strength *s*_*i*_, which corresponds to a node’s actual resource. For this purpose, we first focus on quantity, which indicates the connectivity of a node to other nodes with one degree of separation^11^:

1 $${r}_{i}\equiv \frac{{s}_{i}}{{k}_{i}}\,(\,=\,{r}_{i}^{[1]}).$$

Here, *k*_*i*_ is the degree of node *i*, which is defined as the total number of edges connected to it^10^; i.e.,

2 $${k}_{i}\equiv \sum _{j}{a}_{ij},$$

and *s*_*i*_ is the strength of node *i*, which is defined as the sum of the weights of all the edges connected to it^7,8^, namely,

3 $${s}_{i}\equiv \sum _{j}{w}_{ij}.$$

Strength is interpreted as a resource assigned to each node. For example, in financial networks, it represents the wealth of each individual; in scientific collaboration networks, it represents the number of the papers published by each researcher. In human communication networks, it denotes the total communication time on a given day spent by all individuals communicating with each individual^6,7^. Thus, ${r}_{i}^{[1]}$ means the average level of resource of all nodes directly connected to node *i*.

### Node Property of Connectability to Other Nodes Within Two Degrees of Separation

In this study, to define new centrality of weighted networks of connectability of each node to others within a range of two degrees of separation, we extend ${r}_{i}^{[1]}$ as follows:

4 $${r}_{i}^{[2]}\equiv \frac{{s}_{i}}{{k}_{i}^{[2]}},$$

where ${k}_{i}^{[2]}$ represents the number of nodes connectable to node *i* within two degrees of separation from node *i*, defined as:

5 $${k}_{i}^{[2]}\equiv |\{\,j\,|\,l(i,\,j)\le 2,\,j\ne i\}|.$$

**Figure 2:**
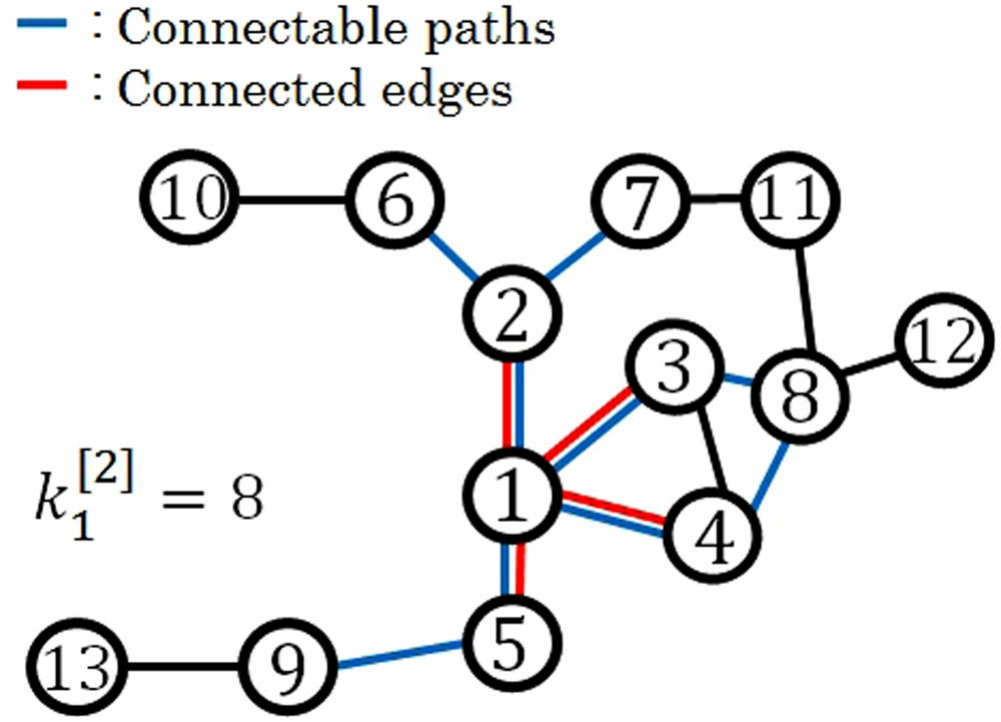
Calculated example of ${k}_{i}^{[2]}$. This is a part of a weighted network, which is an area mainly composed of the nodes within two degrees of separation from node 1. In this case, the nodes counted in ${k}_{1}^{[2]}$ are shown as those connected by blue edges, except node 1. In addition, the red paths represent the edges directly connected to node 1. The number of the nodes within two degrees of separation of node 1, ${k}_{1}^{[2]}=|\{\,j\,|\,l(1,\,j)\le 2,\,j\ne 1\}|$ $=|\{2,\,3,\,4,\,5,\,6,\,7,\,8,\,9\}|=8$.

Here, $|\,\cdot \,|$ represents a cardinal number of a set $\{\,\cdot \,\}$, and *l*(*i*, *j*) represents the smallest number of steps from node *i* to node *j*. {*j*|*l*(*i*, *j*) ≤ 2, *j* ≠ *i*} represents the set of nodes within two degrees of separation of node *i*. Therefore, ${k}_{i}^{[2]}$ represents the number of nodes with two degrees of separation from node *i*. Note that the nodes correspond to the set {*j*|*l*(*i*, *j*) ≤ 2, *j* ≠ *i*}, which includes the nodes directly connected with node *i*, i.e., the set includes the region within one degree of separation. This is because we consider not only the nodes within two degrees of separation but also actual connected nodes to be the connectable nodes of node *i*. Fig. 2 shows a calculated example of ${k}_{i}^{[2]}$. This is a part of a weighted network, which is an area mainly composed of the nodes within two degrees of separation from node 1. In this case, the nodes counted in ${k}_{1}^{[2]}$ are shown as all nodes attached by blue edges except node 1. According to equation (5), {*j*|*l*(1, *j*) ≤ 2, *j* ≠ 1} = {2, 3, 4, 5, 6, 7, 8, 9}. Therefore, the number of nodes connectable to node 1 is ${k}_{1}^{[2]}=|\{2,\,3,\,4,\,5,\,6,\,7,\,8,\,9\}|=8$. In addition, *s*_1_ is calculated based on the weight of the directly connected nodes 2, 3, 4, and 5. ${r}_{1}^{[2]}$ represents the average resource of node 1 from the nodes within two degrees of separation, which is the allocable resource of node 1. In general, it is considered that the more ${r}_{i}^{[2]}$ increases, the greater is the degree of connectability of node *i* to the range.

In our analysis, we also used a scatter diagram of ${s}_{i}\,vs.\,{r}_{i}^{[2]}$ to investigate global trends in weighted networks through the relationship between the actual resource (*s*_*i*_) and the allocable resource (${r}_{i}^{[2]}$).

### Organizations for Analysis

To validate the usefulness of the centrality ${r}_{i}^{[2]}$, it was applied to face-to-face communication networks in two corporate organizations. The networks were constructed from time-series data from face-to-face contact events of employees in the organizations. In each organization, employees had attached a wearable device, called a Business Microscope (Hitachi, Ltd, Japan)^18^, during working hours for the measurement period. The data were collected by this device to one minute of temporal resolution, and were provided by the World Signal Center, Hitachi, Ltd., Japan (see Supplementary Information 1). In each network, the weight of the connection between nodes (employees) corresponds to the communication time [in minutes] of each pair.

Table 1 shows information about two corporate organizations (A and B) for analysis. In this table, ‘Type’ denotes the job categories of each organization. ‘Participants’ denotes the number of the employees who communicate with each other during the measurement period. ‘Days’ denotes the measurement period without Saturdays, Sundays, and holidays. Furthermore, organization A has two departments: Research & Development and Administration, whereas organization B has three departments: Product Development, Sales, and Administration. The numbers of employees in the departments of organization A were 137 and 21, respectively, with 140, 42, and 29 in organization B, respectively.

**Table 1:** Information about two corporate organizations.

Type	Organization A	Organization B
	Research & Development	Wholesale
Participants	158	211
Days	43	47
Departments	2	3

### Node Centrality in Face-to-Face Communication Networks

Table 2 shows the top 10 nodes ranked according to centralities ${r}_{i}^{[2]}$, ${r}_{i}^{[1]}$, and *s*_*i*_ in organization A. In this table, 50% of the employees identified by the new centrality ${r}_{i}^{[2]}$ differ from those identified by *s*_*i*_ as a reference, while 40% of the employees identified by the previous centrality ${r}_{i}^{[1]}$ differ from those identified by the reference centrality *s*_*i*_. Furthermore, 50% of the employees identified by the new centrality ${r}_{i}^{[2]}$ differ from those identified by the previous centrality ${r}_{i}^{[1]}$.

**Table 2:** Node centrality in face-to-face communication networks in organization A.

Rank	Node number		
	${{\boldsymbol{r}}}_{{\boldsymbol{i}}}^{{\boldsymbol{[}}{\bf{2}}{\boldsymbol{]}}\,}$	${{\boldsymbol{r}}}_{{\boldsymbol{i}}}^{{\boldsymbol{[}}{\bf{1}}{\boldsymbol{]}}\,}$	${{\boldsymbol{s}}}_{{\boldsymbol{i}}}$
1	6	6	6
2	9	118	71
3	71	113	118
4	149	71	20
5	31	32	76
6	32	121	63
7	4	104	4
8	29	4	113
9	15	20	3
10	3	31	9

Table 3 shows the top 10 nodes ranked according to each centrality in organization B. In this table, 70% of the employees identified by the new centrality ${r}_{i}^{[2]}$ differ from those identified by the reference centrality *s*_*i*_, while 40% of those identified by the previous centrality ${r}_{i}^{[1]}$ differ from those identified by reference centrality *s*_*i*_. Moreover, 60% of the employees identified by the new centrality ${r}_{i}^{[2]}$ differ from those identified by the previous centrality ${r}_{i}^{[1]}$. We also note a remarkable trend whereby all of the top 23 employees identified by the new centrality ${r}_{i}^{[2]}$ belong to the same department, Administration. No such trend was identified by the other centralities.

**Table 3:** Node centrality in face-to-face communication networks in organization B.

Rank	Node number		
	${{\boldsymbol{r}}}_{{\boldsymbol{i}}}^{{\boldsymbol{[}}{\bf{2}}{\boldsymbol{]}}\,}$	${{\boldsymbol{r}}}_{{\boldsymbol{i}}}^{{\boldsymbol{[}}{\bf{1}}{\boldsymbol{]}}\,}$	${{\boldsymbol{s}}}_{{\boldsymbol{i}}}$
1	190	197	197
2	203	196	123
3	202	190	196
4	201	142	120
5	195	141	190
6	209	203	126
7	199	193	193
8	205	194	203
9	211	123	62
10	193	202	50

These results show the possibility that investigating the allocable weights of two degrees of separation by ${r}_{i}^{[2]}$ can distinguish social role more accurately than ${r}_{i}^{[1]}$ and s_*i*_, which correspond to actual resources.

We also analyse the average values of ${r}_{i}^{[2]}$ and typical centrality measures (degree *k*_*i*_, strength *s*_*i*_, clustering coefficient *c*_*i*_, Barrat’s weighted clustering coefficient ${c}_{i}^{w}$, closeness centrality *cc*_*i*_, weighted closeness centrality $c{c}_{i}^{w}$, betweenness centrality *bc*_*i*_, and weighted betweenness centrality $b{c}_{i}^{w}$ and ${r}_{i}^{[1]}$) in each department in organization B^1,7–9,11^ (see Supplementary Table S1). Supplementary Table S1 shows that the average values of our new centrality in Administration are about six times greater than the average value of the other departments. This large difference in the average value of the departments cannot be observed in the other departments. This result suggests that our new centrality can detect this kind of subgroup more clearly and sensitively than the other typical centrality measures.

### Global Trends in Face-to-Face Communication Networks

**Figure 3:**
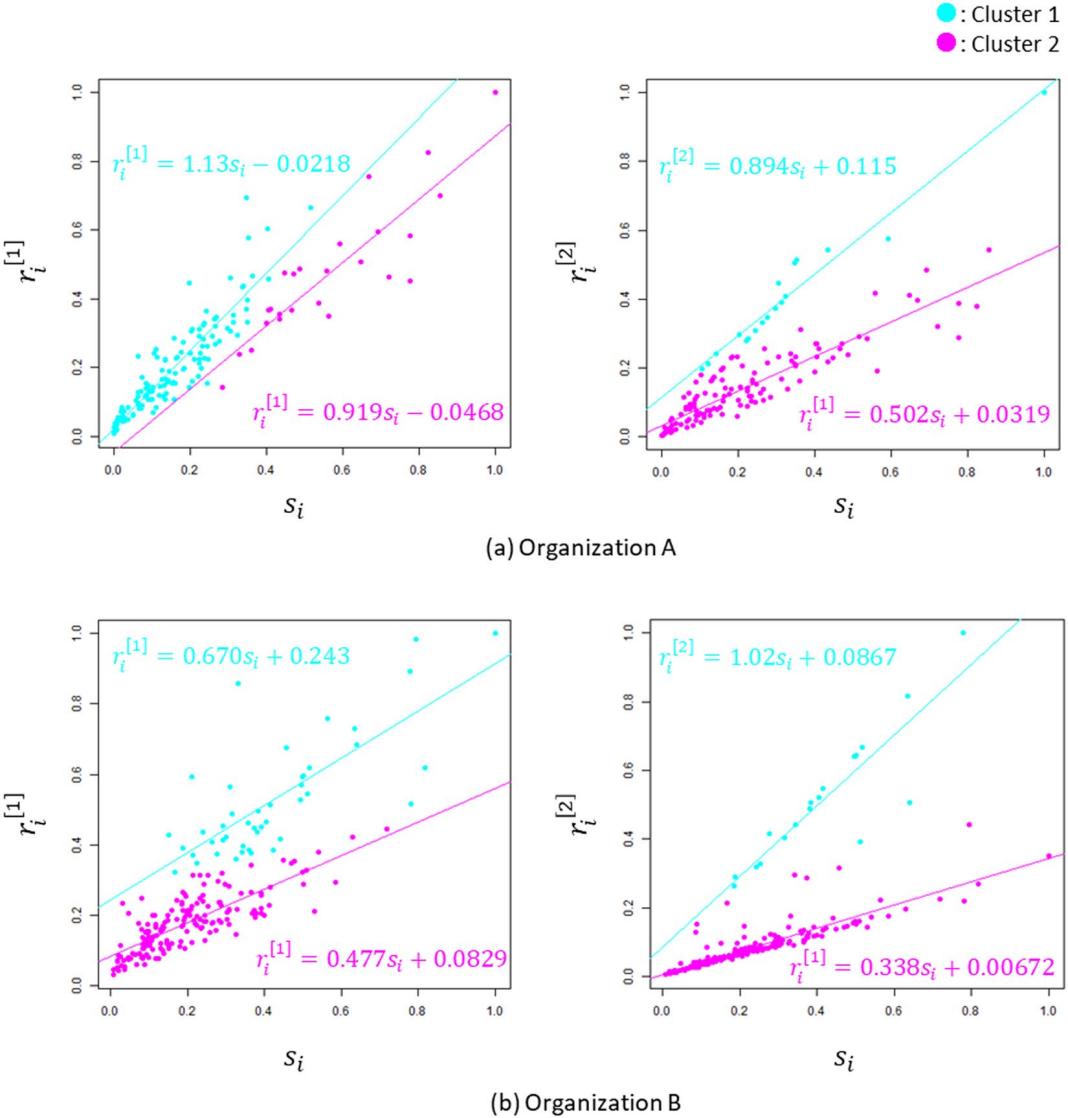
The scatter diagrams ${s}_{i}\,vs.\,{r}_{i}^{[1]}$ and ${s}_{i}\,vs.\,{r}_{i}^{[2]}$. (**a)** and (**b)** illustrate the scatter diagrams ${s}_{i}\,vs.\,{r}_{i}^{[1]}$ and ${s}_{i}\,vs.\,{r}_{i}^{[2]}$ of organizations A and B, respectively, in which the straight lines are the regression lines for the clusters detected using PAM. The two clusters are shown by the light blue plot and the purple plot in each scatter diagram. Each plot in these scatter diagrams is normalized according to the maximum value of each centrality.

Fig. 3a and b illustrate the scatter diagrams ${s}_{i}\,vs.\,{r}_{i}^{[1]}$ and ${s}_{i}\,vs.\,{r}_{i}^{[2]}$ of the organizations A and B, respectively, in which the straight lines are the regression lines for the clusters described below. In this analysis, the *P* values for the slopes of all regression lines are statistically significant (*P* < 0.001). Each plot in these scatter diagrams is normalized according to the maximum value of each centrality. Here, the plots on the scatter diagrams ${s}_{i}\,vs.\,{r}_{i}^{[2]}$ seem to have separated into linear patterns. To cluster the plots objectively, based on such linear correlations, the Mahalanobis generalized distance measure is expected to perform well. Therefore, we use the Partitioning Around Medoids (PAM) method, which can employ the Mahalanobis generalized distance as a measure of dissimilarity, for all scatter diagrams (see the Methods section). As a result, two clusters are shown as light blue plots and purple plots in each scatter diagram. In organization B, the proportion of the cluster shown as light blue plots (cluster 1) formed by Administration is 65.5%, and purple plots (cluster 2) are constructed by 35.5% of Administration and Product Development, Sales in the scatter diagram of ${s}_{i}\,vs.\,{r}_{i}^{[2]}$.

The scatter diagrams of ${s}_{i}\,vs.\,{r}_{i}^{[1]}$ of both organizations show a linear increasing trend in ${r}_{i}^{[1]}$ with similar slopes with respect to *s*_*i*_ in the two clusters. In contrast, the scatter diagrams of ${s}_{i}\,vs.\,{r}_{i}^{[2]}$ of both organizations show that the regression line of the light blue cluster has a high value (0.894 or 1.02) for slope while that of the purple cluster has a low value (0.502 or 0.338); hereafter these will be called ‘trend 1’ and ‘trend 2’.

**Figure 4:**
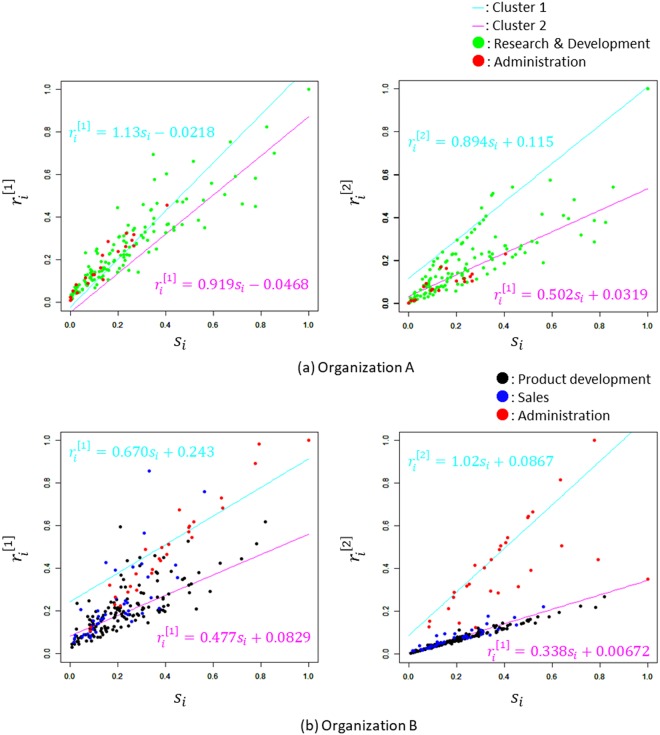
The scatter diagrams ${{s}}_{{\boldsymbol{i}}}\,{vs}.\,{{r}}_{{\boldsymbol{i}}}^{[1]}$ and ${{s}}_{{i}}\,{vs}.\,{{r}}_{{i}}^{[2]}$ show the information on the affiliated departments. (**a**) and (**b**) provide different information on the same scatter diagrams, ${s}_{i}\,vs.\,{r}_{i}^{[1]}$ and ${s}_{i}\,vs.\,{r}_{i}^{[2]}$, of organizations A and B as shown in Fig. 3a and b, in which the job types of the employees are shown in different colours. In organization A, the employees belonging to Research & Development and Administration are represented by green and red plots, respectively. In organization B, the employees belonging to Product Development, Sales, and Administration are represented by black, blue, and red plots, respectively. The proportion of the cluster shown as light blue plots (cluster 1) formed by Administration is 65.5%, and purple plots (cluster 2) are constructed by 35.5% of Administration and Product Development, Sales in the scatter diagram of ${s}_{i}\,vs.\,{r}_{i}^{[2]}$.

Fig. 4a and b provide different information on the same scatter diagrams, ${s}_{i}\,vs.\,{r}_{i}^{[1]}$ and ${s}_{i}\,vs.\,{r}_{i}^{[2]}$, of organizations A and B as shown in Fig. 3a and b, in which the job types of the employees are shown in different colours. In the scatter diagrams of ${s}_{i}\,vs.\,{r}_{i}^{[1]}$ for organizations A and B, the correspondence between the regression lines and the job types is unclear. The same is true in ${s}_{i}\,vs.\,{r}_{i}^{[2]}$, the scatter diagram for organization A. However, that for organization B shows clear correspondence between the regression lines and the job types. Specifically, all of the 19 employees classified as trend 2 entirely belong to Administration. Conversely, most of the 192 employees classified as trend 1 belong to Product Development or Sales.

In addition, we analyse typical global weighted centrality measures, weighted closeness centrality $c{c}_{i}^{w}$, and weighted betweenness centrality $b{c}_{i}^{w}$. In particular, we visualize the scatter diagrams ${s}_{i}\,vs.\,c{c}_{i}^{w}$ and ${s}_{i}\,vs.\,b{c}_{i}^{w}$ (see Supplementary Figures S1 and S2). The results indicate the linear increasing trends observed in the scatter diagram ${s}_{i}\,vs.\,{r}_{i}^{[2]}$, which is an inherent trend that can only be obtained by ${r}_{i}^{[2]}$ in the face-to-face communication network.

## Discussion

The measures of node centrality shown in Table 2 and Table 3 suggest that the new centrality ${r}_{i}^{[2]}$ provides a new property that is different from those represented by the previous centralities ${r}_{i}^{[1]}$ and s_*i*_. In particular, we consider that the new centrality ${r}_{i}^{[2]}$ represents involvedness as an aspect of connectability or a node’s allocable resource. Specifically, ${k}_{i}^{[2]}$ represents the number of the nodes connectable to node *i* within two degrees of separation. Thus, in face-to-face communication networks, the more ${k}_{i}^{[2]}$ decreases, the fewer employees there are who can communicate with employee *i*. Moreover, *s*_*i*_ represents the total communication time of employee *i* with other employees. Thus, the more *s*_*i*_ increases, the more employees there are who can communicate with employee *i* or the more communication time employee *i* has with specific employees.

Thus, if ${r}_{i}^{[2]}$ increases, either the number of employees connectable to employee *i* within a range of two degrees of separation decreases, or the communication time of employee *i* with a specific employee increases. Therefore, ${r}_{i}^{[2]}$ provides employee *i*’s possible connection strength for easily connectable employees, who are in the range of two degrees of separation. In other words, the new centrality ${r}_{i}^{[2]}$ can be intuitively considered a measure of involvedness in communication by employee *i*. In the case of organization B, Administration is in a high-involvedness communication environment compared with the other departments.

Fig. 3a and b show that in the scatter diagram of ${s}_{i}\,vs.\,{r}_{i}^{[1]}$, the employees of both organizations were divided into two clusters with different trends. Furthermore, Fig. 4a and b show that the two clusters correspond well with the job types in organization B. These results suggest that the scatter diagram of ${s}_{i}\,vs.\,{r}_{i}^{[2]}$ can detect subgroups corresponding to collective functions that cannot be detected by the scatter diagram of ${s}_{i}\,vs.\,{r}_{i}^{[1]}$.

In the employees corresponding to trend 1 in each organization, those who spend much time in face-to-face communication have the opportunity for prolonged communication with employees who can easily connect to employee *i* (high involvedness). Indeed, in organization B, the employees on trend 1 are intuitively expected to follow the communication trend peculiar to clerical work, which offers opportunities for intimate and private communication. In contrast, the employees corresponding to trend 2 in each organization are thought to work in an open environment that facilitates brief communication with other employees regardless of face-to-face time (low involvedness). The employees on trend 2 are intuitively expected to show a communication trend peculiar to on-site and standing work, which provides opportunities for open communication. From this point of view, the trend obtained by the scatter diagram of ${s}_{i}\,vs.\,{r}_{i}^{[2]}$ is expected to correspond to social roles.

${r}_{i}^{[2]}$ represents node *i*’s allocable resource for the nodes within two degrees of separation of node *i*. By introducing this centrality, we can quantify potential ability for local structural formation of each node, which is not explicitly targeted by conventional centrality. In other words, ${r}_{i}^{[2]}$ quantifies not an actual network structure of each node but its potential connectability. For example, we can answer the question of how deeply and narrowly node *i* can construct connections with other nodes by applying the scatter diagram of ${s}_{i}\,vs.\,{r}_{i}^{[2]}$. Thus, the proposed method is expected to be effective in analyses of the network in which each node allocates its limited resource, for example, with respect to friends/acquaintance, power grid, transportation, and so on. Furthermore, ${r}_{i}^{[2]}$ can provide the criterion for the dynamics of weighted networks. Various mathematical models of network dynamics have been proposed for explaining real phenomena, such as power law correlation s~*k*^*θ*^ ^13–16^. In this regard, our proposed centrality ${r}_{i}^{[2]}$ may offer a viewpoint based on the possible resource allocation for a mathematical model of the temporal development of weighted network structure.

In equation (5), we assume a very simple situation wherein all of the nodes are within a range of two degrees of separation from node *i*. However, realistic cases in which triadic closures do not occur also exist. Therefore, as a remaining problem, we should consider this case in ${r}_{i}^{[2]}$ by introducing a new parameter. In addition, the above results provide a fresh perspective that calls attention to the effect of middle-range structure among agents in a social network, in a broad sense. Hence, developing a new centrality by which we can set and analyse an arbitrary middle range from each node also remains a problem. If we introduce the new centrality, it is expected to reveal the distance to the effective cut-off from each node for the characterization of that node and a social network.

## Methods

### Clustering the Scatter Diagrams

In the analysis of the scatter diagram ${s}_{i}\,vs.\,{r}_{i}^{[2]}$ in Fig. 3b, the PAM for the k-medoids method was used for clustering the scatter plots^19^. In PAM, to highlight the correlation of point sequences, the squared Mahalanobis generalized distance of vector ***x*** with respect to an average vector ***μ*** and a covariance matrix Σ

6 $${{\boldsymbol{D}}}^{2}={({\boldsymbol{x}}-{\boldsymbol{\mu }})}^{T}{{\rm{\Sigma }}}^{-1}({\boldsymbol{x}}-{\boldsymbol{\mu }})$$

was used as a measure of dissimilarity in computing the dissimilarity matrix. In PAM, the number of clusters was set to *k* = 2.

## Electronic supplementary material

Supplementary information

Instructions of supplementary dataset

Dataset 1

Dataset 2

Dataset 3

Dataset 4
