# Author Correction: Node property of weighted networks considering connectability to nodes within two degrees of separation

**DOI:** 10.1038/s41598-018-30955-z

**Published:** 2018-08-20

**Authors:** Shun-ichi Amano, Ken-ichiro Ogawa, Yoshihiro Miyake

**Affiliations:** Department of Computer Science, Tokyo Institute of Technology, Yokohama, Kanagawa Japan

Correction to: *Scientific Reports* 10.1038/s41598-018-26781-y, published online 31 May 2018

In the original version of this article, there was a typographical error in the spelling of the author Shun-ichi Amano which was incorrectly given as Sun-ichi Amano.

In addition, Reference 11 was incorrectly given as: ‘Sun, X., Shen, H., Cheng, X. & Wang, Z. Degree-strength correlation reveal anomalous trading behaviour. *PLoS One* **7**, e45598 (2012).’

These errors have now been corrected in the HTML and PDF versions of the Article.

