# Supplementary material for: Node property of weighted networks considering connectability to nodes within two degrees of separation
Source: Sci Rep. 2018 May 31;8:8464. doi: 10.1038/s41598-018-26781-y (PMC5981652; doi:10.1038/s41598-018-26781-y)
Supplement: Supplementary file 1 — Supplementary information [file 41598_2018_26781_MOESM1_ESM.docx]

Node property of weighted networks considering connectability to nodes within two degrees of separation

***Shun-ichi Amano^1^, Ken-ichiro Ogawa^1^, and Yoshihiro Miyake^1^***

^1^ Department of Computer Science, Tokyo Institute of Technology, Yokohama, Kanagawa, Japan.

Correspondence and requests for materials should be addressed to S.A. (email: Amano@myk.dis.titech.ac.jp)

**Supplementary Table S1. The average values of each centrality for each department in organization B.**


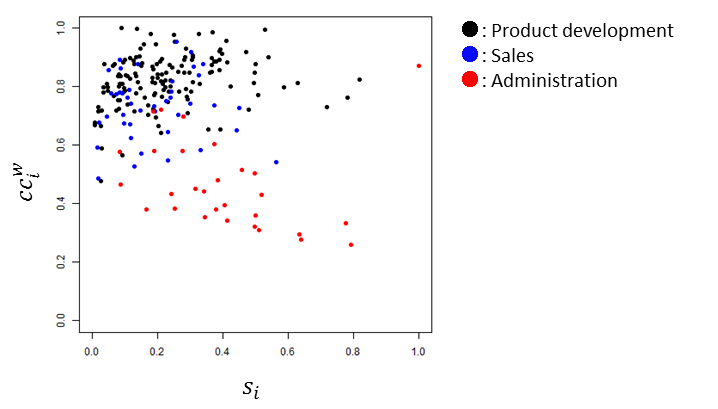


| Organization B | $k$ | $s$ | $c$ | $c^{w}$ | $cc$ | ${cc}^{w}$ | $bc$ | ${bc}^{w}$ | $r^{[1]}$ | $r^{[2]}$ |
| --- | --- | --- | --- | --- | --- | --- | --- | --- | --- | --- |
| All | 31.28 | 1054.9 | 0.5348 | 0.6138 | 0.0022 | 0.0012 | 125.6 | 269.48 | 34.649 | 9.5449 |
| Product development | 33.971 | 983.75 | 0.4729 | 0.5635 | 0.0023 | 0.0013 | 125.91 | 252.51 | 28.52 | 5.8831 |
| Sales | 26.286 | 820.24 | 0.5088 | 0.5911 | 0.0022 | 0.0011 | 108.94 | 203.3 | 32.737 | 5.6825 |
| Administration | 25.517 | 1738.2 | 0.8895 | 0.0018 | 0.0018 | 0.0007 | 148.19 | 447.3 | 67.007 | 32.816 |

**Supplementary Figure S1. Scatter diagram** $s_{i} vs. {cc}_{i}^{w}$ **with the information of the affiliation departments in organization B.** The employees belonging to Product development, Sales, and Administration are represented by the black plots, the blue plots, and the red plots, respectively. The red plots tend to be located below the other plots. Thus, weighted closeness centrality can detect Administration in organization B. However, the inherent trend in each department is unclear. In other words, weighted closeness centrality can’t clearly detect the linear trends of each department seen in the scatter diagrams of $s_{i} vs. r_{i}^{[2]}$, in organization B.


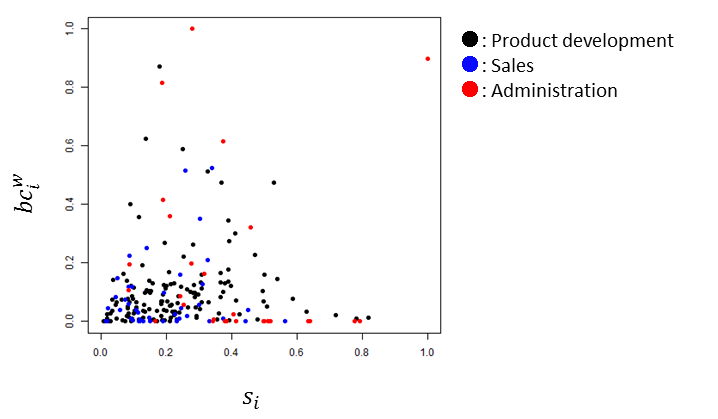


**Supplementary Figure S2. Scatter diagram** $s_{i} vs. {bc}_{i}^{w}$ **with the information of the affiliation departments in organization B.** The employees belonging to Product development, Sales, and Administration are represented by the black plots, the blue plots, and the red plots, respectively. The plots of each department doesn’t show any specific trends. In addition, the plots of each department are mixed each other. Thus, weighted betweeness centrality can’t clearly detect Administration and the inherent trends in each department, in organization B.
