# Supplementary material for: Node property of weighted networks considering connectability to nodes within two degrees of separation
Source: Sci Rep. 2018 May 31;8:8464. doi: 10.1038/s41598-018-26781-y (PMC5981652; doi:10.1038/s41598-018-26781-y)
Supplement: Supplementary file 2 — Instructions of supplementary dataset [file 41598_2018_26781_MOESM2_ESM.docx]

There are four EXCEL files: “Adj_OrgA.xlsx”, “Adj_OrgB.xlsx”, “List_OrgA.xlsx”, and “List_OrgB.xlsx”. “Adj_OrgA.xlsx” and “Adj_OrgB.xlsx” are the adjacency matrix of Organization A and Organization B, respectively. “List_OrgA.xlsx” and “List_OrgB.xlsx” are the list which show the correspondence between each node number and its affiliation department in Organization A and Organization B, respectively. Note here that the node numbers are not same as the column (row) number of the adjacency matrix in “Adj_OrgA.xlsx”, “Adj_OrgB.xlsx”. So, both the node number and the column (row) number of the adjacency named “Adj” are shown in the lists. By this, you can confirm the correspondence between both number series.
